# Epidermal Hyaluronan in Barrier Alteration-Related Disease

**DOI:** 10.3390/cells10113096

**Published:** 2021-11-09

**Authors:** Céline Evrard, Catherine Lambert de Rouvroit, Yves Poumay

**Affiliations:** Research Unit for Molecular Physiology (URPhyM), Department of Medicine, Namur Research Institute for Life Sciences (NARILIS), University of Namur, B-5000 Namur, Belgium; celine.evrard@unamur.be (C.E.); catherine.lambert@unamur.be (C.L.d.R.)

**Keywords:** hyaluronan, epidermal extracellular matrix, atopic dermatitis

## Abstract

In skin, although the extracellular matrix (ECM) is highly developed in dermis and hypodermis, discrete intercellular spaces between cells of the living epidermal layers are also filled with ECM components. Herein, we review knowledge about structure, localization and role of epidermal hyaluronan (HA), a key ECM molecule. HA is a non-sulfated glycosaminoglycan non-covalently bound to proteins or lipids. Components of the basal lamina maintain some segregation between the epidermis and the underlying dermis, and all epidermal HA is locally synthesized and degraded. Functions of HA in keratinocyte proliferation and differentiation are still controversial. However, through interactions with partners, such as the TSG-6 protein, HA is involved in the formation, organization and stabilization of the epidermal ECM. In addition, epidermal HA is involved in the formation of an efficient epidermal barrier made of cornified keratinocytes. In atopic dermatitis (AD) with profuse alterations of the epidermal barrier, HA is produced in larger amounts by keratinocytes than in normal skin. Epidermal HA inside AD lesional skin is located in enlarged intercellular spaces, likely as the result of disease-related modifications of HA metabolism.

## 1. Introduction

Hyaluronan, also called hyaluronic acid (HA), is a linear non-sulfated glycosaminoglycan (GAG) present in extracellular matrices (ECM) of vertebrates [1]. In a 70 kg human body, HA represents around 15 g [2]. First identified in the vitreous humor, HA is also present in skin, muscles, skeleton, and synovial fluid where it plays a role in shock absorption. The skin contains 50% of total HA [3,4]. While the majority of cutaneous HA is localized in the dermis, a significant amount is found between keratinocytes of basal and spinous layers of epidermis [5]. In the epidermis, HA is involved in the establishment of a competent barrier and plays controversial roles in keratinocyte proliferation and differentiation. Some of these functions are made possible by its binding to the membrane receptor CD44 (see Section 3.5 below for further details on CD44) [6]. Other protein partners are involved in HA functions and ECM organization. In particular, the TSG-6 protein appears to be essential for the retention of HA in the epidermal ECM and its organization. The importance of HA in the skin has been reviewed by Kavasi et al., 2017 [7], and Muto et al., 2019 [8]. The present review focuses on HA in the epidermal extracellular matrix and its potential implication in atopic dermatitis, a disease associated with an altered epidermal barrier. 

## 2. The Epidermal Extracellular Matrix

**Figure 1:**
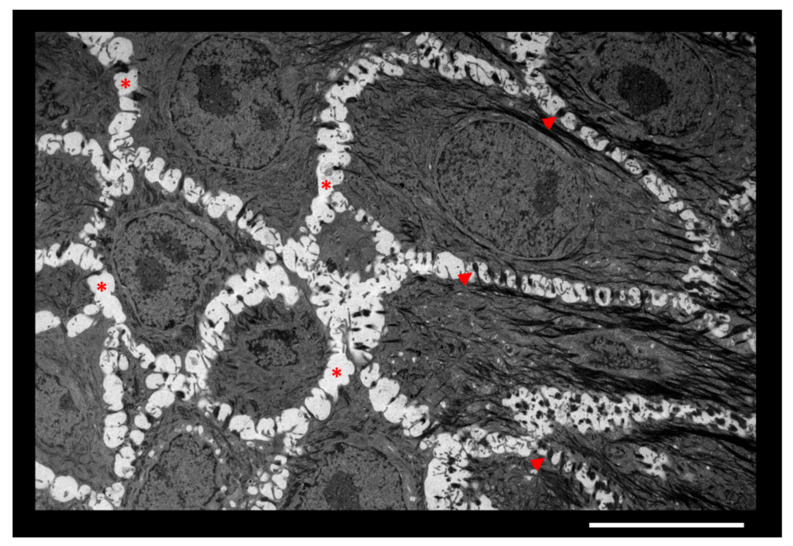
Intercellular spaces between keratinocytes of epidermis. Granular layer of an in vitro reconstructed human epidermis observed by transmission electron microscopy. Adjacent keratinocytes are separated by ECM (*) and anchored to each other by desmosomes (arrowheads). Scale bar = 10 µm.

In skin, the ECM is most abundant in dermis and hypodermis. However, a discrete ECM is, nonetheless, present in the epidermis between cells of the living basal, spinous, and granular layers, where keratinocytes are separated by intercellular spaces approximately 15–20 nm thick. Thereby, the epidermal ECM can solely be directly observed by transmission electron microscopy (Figure 1).

The sugar-rich composition of epidermal ECM was first revealed in 1951 using Alcian blue staining [9]. The epidermal ECM is accordingly largely composed of carbohydrate macromolecules called glycosaminoglycans (GAGs). Among GAGs, those with covalent link to serine residues in core-proteins give rise to proteoglycans. Heparan sulfate and chondroitin sulfate, for instance, account, respectively, for 60% and 20% of such GAGs in the epidermis [10], where they participate in formation of proteoglycans, such as glypican, syndecans (1 to 4), and versican [11,12,13]. Hyaluronan, the only non-sulfated GAG and the only GAG without link to proteins, is also found in the epidermal ECM [5]. An in vitro study of human keratinocytes has shown that HA accounts for 54% of GAGs released in culture medium by this cell type [14]. In human skin, the epidermal ECM contains 25 µg of HA/g of fresh epidermis, while the dermis contains between 120 and 200 µg of HA/g of fresh dermis [3,15]. Together, GAG and proteoglycans create a specialized intercellular environment favorable to the diffusion of nutrients, growth factors, and cytokines [12,16], as well as for eventual upcoming immune cells. Furthermore, some types of chondroitin sulfate proteoglycans (e.g., MCSP) are expressed in an age-dependent manner by epidermal cells that exhibit stem cell properties [17].

The ECM can be divided into two zones: the pericellular matrix and the intercellular matrix. The pericellular matrix is in close contact with neighboring cells, allowing direct interaction with cellular receptors [18]. In pericellular matrix, HA can interact with its trans-membrane receptor CD44, and cytokines and growth factors can rapidly bind their receptors. The intercellular matrix is found between cells without direct contact [18]. In the epidermis, intercellular spaces are very thin. Nevertheless, they can increase in certain pathologies, such as atopic dermatitis, which is characterized by an increased epidermal HA production able to retain a large amount of water, thereby creating spongiosis [19]. These increased spaces notably promote the migration of immune cells into the epithelial tissue.

## 3. Epidermal Hyaluronan

### 3.1. Molecular Structure of HA

HA is composed of repeated disaccharide units made of D-glucuronic acid (GlcUA) and D-N-acetylglucosamine (GlcNAc). These saccharides are bound by β-1.3 and β-1.4 linkages to form long linear polymers [β1.4-GlcUA-β1.3-GlcNAc-]_n_ of high molecular weights, usually ranging from 10^5^ to 10^7^ Daltons, and from 2 to 25 µm in length [20,21]. HA polymers interact with proteins [3], although they never bind through covalent link. Specific HA-interacting proteins are called hyaladherins or HA-binding proteins (HABP). Some of them, such as CD44 and RHAMM, are membrane-bound, and recognized as two HA receptors on cell plasma membrane. Other hyaladherins are secreted into the ECM, such as the TNFα-stimulated-gene-6 (TSG-6) protein involved in extracellular cross-linking of HA [22]. Some proteoglycans, such as versican, are also able to bind HA via their N-terminal domain, thereby creating complex molecular networks in the ECM [2,16,23]. Moreover, since HA carries negative charges, this macromolecule can retain a large amount of water (up to 70% of its weight) through its remarkable hydrophilicity. Such characteristics of HA confer viscoelastic properties to the HA-containing tissues and spaces [4,21].

### 3.2. Synthesis of HA

HA is synthesized by enzymes located at the inner side of the plasma membrane and simultaneously exported as polymers directly in the ECM. These enzymes, called HA synthases (HAS), are glycosyltransferases [24]. The substrates utilized by HAS enzymes are UDP-α-N-acetyl-D-glucosamine (UDP-GlcNAc) and UDP-α-D-glucuronate (UDP-GlcUA) originating from cellular metabolism of glucose [25]. In mammals, three HAS isoenzymes have been identified: HAS1, HAS2, and HAS3. Encoded by genes located on different chromosomes (19q13.3 for *HAS1*, 8q24.12 for *HAS2* and 16q22.1 for *HAS3*), HAS enzymes share 55 to 70% identity [26]. They contain, between N-terminal and C-terminal regions, a central region harboring 75–87% similarity [27]. All three enzymes have seven transmembrane domains [24] and have distinct binding domains for the two substrates [25]. Although structurally similar, the three HAS enzymes are characterized by very different temporal expression patterns, by the sizes of generated HA polymers, and by different enzymatic activities [27]. HAS activity is partially dependent on the concentration of available cellular UDP-GlcNAc. While their V_max_ do not show any significant differences, the K_m_ of HAS1 enzyme is more elevated than the K_m_ of HAS2 and HAS3 enzymes, showing that HAS1 exhibits a lower affinity for its substrate than HAS2 and HAS3 [27]. Consequently, HAS1 enzyme is unable to form HA polymers if the amount of available UDP-GlcNAc is low. On the other hand, the activity of HAS2 appears poorly influenced by substrate concentration, and the activity of HAS3 remains independent of UDP-GlcNAc concentration [28].

*HAS1* mRNA is expressed during early stages of embryonic development (gastrulation and neurulation) and during cell differentiation. This synthase has a low enzymatic activity since the amount of product formed per minute is low. HAS1 enzyme uses large amounts of UDP-GlcNAc, allowing the production of high molecular weight HA polymers [27,28]. In the epidermis, an in vitro study showed that *HAS1* mRNA is expressed during keratinocyte differentiation [29]. HAS2 enzyme is essential during embryonic development [30]. While *HAS2* mRNA is very weakly expressed in adult tissues, its overexpression causes skin changes. In particular, *Has2* mRNA overexpression induces an increased amount of HA with increased thickness of the skin, as in the skin of the Sharpei dog breed [31]. Similarly, naked mole rats carry mutations in *Has2* gene that cause an abnormally enhanced synthesis of high molecular weight HA [32]. Conversely, in elderly humans, the typical thinning of the skin is accompanied by decreased *HAS2* mRNA expression [33]. HAS2 is characterized by a slightly higher activity than HAS1 and generates in vitro HA polymers of comparable molecular weights. However, HA polymers generated by HAS2 in vivo in transfected cells exhibit higher molecular weights than those made by HAS1 [27]. HAS3 is the most active enzyme and synthesizes, therefore, low molecular weight polymers (100–1000 kDa) [27]. This enzyme is predominantly expressed in inflammatory processes (reviewed in Tavianatou et al., 2019 [34]), including inflamed skin [35]. Accordingly, Malaisse et al. (2014) [29] highlighted that *HAS3* mRNA is overexpressed in lesional atopic dermatitis skin, while *HAS1* mRNA was preferentially expressed in healthy epidermis. However, in a different study, divergent data indicate abundant mRNA expression of *HAS3* in healthy keratinocytes [36].

HAS activity can be modulated by growth factors and cytokines, such as keratinocyte growth factor (KGF), epidermal growth factor (EGF), tumor necrosis factor α (TNF-α), transforming growth factor β (TGF-β), and bone morphogenetic protein (BMPs), as well as by extracellular UTP [37,38,39]. Post-translational modifications of HAS enzymes can occur [40]. Phosphorylation [41,42] and O-GlcNAcylation [43] of HAS enzyme increases their activities. However, the link between post-translational modifications and changes in enzymatic activity remains unknown. Finally, some mono-ubiquitination of HAS2 could be involved in its enzymatic activity, as mutated lysine residue 190, the acceptor site for ubiquitin, is responsible for HAS2 enzyme inactivation [44].

### 3.3. Degradation of HA

The half-life of HA is relatively short in the epidermis, ranging from 1 to 2 days [3,45]. Enzymes responsible for HA degradation are endoglycosidases and exoglycosidases. Hyaluronidases (HYAL) are members of the endoglycosidase family and specifically cleave the β-1.4 bonds in HA polymers. The residual oligosaccharides are then hydrolyzed by exoglycosidases into β-D-glucuronate and β-N-acetyl-D-hexosamine [46]. In humans, five hyaluronidase-like genes and one pseudogene (*HYALP1*) have been identified. They share 40% similarity. *HYAL1*, *2*, and *3* are located on chromosome 3p21.3 and have similar genomic structure with four exons. *HYAL4*, *HYAL5 (PH20)*, and *HYALP1* are located on chromosome 7q31.3 and present one extra exon compared to *HYALs* encoded on chromosome 3. Beside the hyaluronidase-like genes, another enzyme implicated in HA catabolism has been identified. *KIAA1199*, also named *CEMIP* (cell-migration inducing protein) or *HYBID* (HA-binding protein involved in HA depolymerization), is located on chromosome 15q25.1 and presents no significant similarity with other hyaluronidases [47]. HYAL1, HYAL2, KIAA1199, and PH20 enzymes are able to cleave HA polymers [47,48]. While PH20 has an activity restricted to spermatozoa, KIAA1199, HYAL1, and HYAL2 are active in various somatic tissues. KIAA1199 enzyme is notably involved in the regulation of infection-related dermis inflammation as its absence has been shown to increase inflammation and antimicrobial activity [49]. It is also implicated in photoaging skin through its action on fibroblasts [50]. It degrades HA by means of endocytosis of clathrin-coated membrane regions [50]. Only HYAL1 and HYAL2 are expressed by keratinocytes [51,52].

Hyaluronidase 1 is responsible for the cleavage of HA polysaccharides into di- or tetra-saccharides [47]. HYAL1 is active in the skin and more specifically in keratinocytes within the granular layer [51]. In terms of subcellular localization, HYAL1 is specifically localized in lysosomes, an acidic compartment (pH about 3.7) necessary for its activity, as the optimal pH for HYAL1 is 3-4 [53,54]. Hyaluronidase 2 is structurally similar to HYAL1 but has a different activity and localization. HYAL2 protein is anchored to the plasma membrane by a glycosylphosphatidylinositol (GPI) anchor and generates fragments of HA around 20kDa of molecular weights [55,56]. HYAL2 can be active at acidic (4) and neutral (7.5) pH [48]. In the epidermis, HYAL2 enzyme is anchored in cell membranes of viable layers [52].

A cellular model of HA degradation based on the involvement of both HYAL1 and HYAL2 enzymes was proposed in the early 2000s [45,57]. Pericellular HA is believed to bind its CD44 receptor at the plasma membrane. HYAL2 protein is also anchored to the cell membrane, close to CD44, creating intimate vicinity between membrane-bound HA and HYAL2 protein, resulting in enzymatic degradation of HA polymers into 20kDa oligosaccharides, which then become internalized in endosomes, along with the enzyme. When HA fragments-containing endosomes fuse with lysosomes, the low pH inside the resulting compartment initiates the HYAL1-driven digestion of HA fragments into di- or tetra-saccharides.

**Figure 2:**
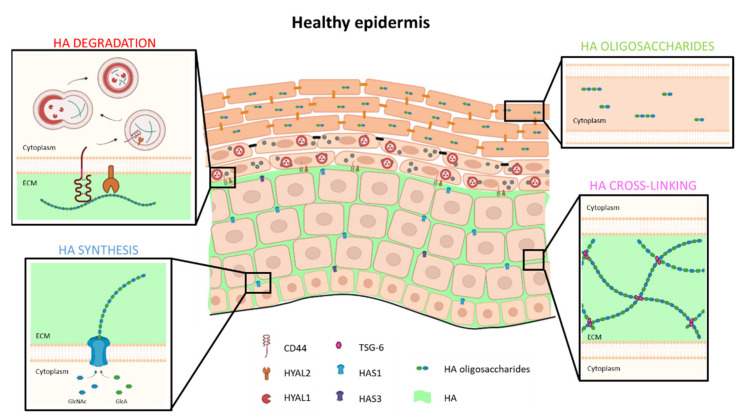
Model of HA metabolism in healthy human epidermis. HA is synthetized and extruded by HAS1 and, to a lesser extent, by HAS3 in ECM of basal and spinous layers. In ECM, TSG-6 protein binds and cross-links HA to trap it locally and form a dense matrix. In the granular layer, extracellular HA binds CD44. Simultaneously, HA is degraded into oligosaccharides by HYAL2 and internalized in endosomes which fuse with lysosomes. At acidic pH of lysosomes, HYAL1 enzyme is active and degrades HA in di- or tetra-saccharides. These low molecular weight HA are then found in corneocytes, where they participate in cutaneous hydration.

At epidermal tissue levels, HA detected using procedures based on HABP appears mainly localized between keratinocytes that constitute basal and spinous layers, but it is not detected in granular and cornified layers [29]. While CD44 and HYAL2 proteins are anchored in the membrane of living layers [52,58], HYAL1 enzyme is active in lysosomal compartment of granular layer [51]. Over the course of differentiation, some residual intracellular HA oligosaccharides seem to end up inside cornified keratinocytes where they most likely play a role in skin hydration together with natural moisturizing factors (NMF) [20,51] (Figure 2). During keratinocyte migration towards the most superficial layers and as a result of the acidification of the upper layers, filaggrin becomes degraded into small free metabolites and amino acids, thereby forming NMF essential to skin hydration, barrier homeostasis and superficial desquamation. Skin hydration is a crucial factor involved in tissue resistance to mechanical stress and contributes to its resilience [59].

One might wonder whether HA present in the ECM of epidermis is exclusively metabolized by keratinocytes or whether exchanges occur between the dermis and the epidermis. Studies based on in vitro reconstructed epidermal models have shown that epidermal HA synthesized by keratinocytes is subsequently found under the epidermal tissue (either in medium [29], or in the supporting collagen matrix [60]), suggesting that HA molecules can escape the epidermis and diffuse into sub-epidermal spaces and, by extension, into the dermis. The skin epidermal-dermal junction is organized by basement membrane (BM) components that create the essential structure between epidermal and dermal HA. Indeed, when the epidermis of elderly people is exposed to sunlight, the BM is somehow damaged by UV exposure, and smaller amounts of HA are observed in exposed epidermis than in unexposed tissue. In addition, the inhibition of metalloproteinases and heparanases, in an in vitro model of full thickness skin equivalent exposed to UV, protects the BM integrity and retains 25% additional HA when compared to HA amount in control epidermis [61]. Consistently, in in vitro rat epidermis model, the setting up of a BM between the epidermis and the collagen matrix was shown to retain approximately 75% HA in the epidermis, whereas 80% of HA was lost in absence of BM [62,63]. In reconstructed human epidermis (RHE) models on polycarbonate filters, cells in the basal layer secrete basement membrane components (lamina densa and lamina lucida) and form hemidesmosomes. Structures organized by RHE on the filter can only be considered as BM-like structures since they lack collagen VII and fibronectin, for instance, in contrast to normal BM [64]. Nevertheless, the proportion of HA retained in RHEs is approximately 95%, since a very small amount of epidermal HA appears released into the underlying medium [29]. In addition to the importance of BM in the retention of epidermal HA, the TSG-6 protein in the extracellular matrix is involved in some cross-linking with HA chains and seems able to prevent HA from “sinking” into the underlying compartment [65] (Figure 2). In summary, a large amount of HA synthesized by keratinocytes does remain in the epidermal matrix and is locally degraded, but about 10% of epidermal HA diffuses into the dermis through the BM and reaches local lymph and blood vessels, in which HA then circulates towards lymph nodes, or the liver, where it is degraded.

### 3.4. Functions of HA in the Epidermis

#### 3.4.1. Regulation of Keratinocyte Proliferation

In keratinocytes, interaction of HA with its CD44 receptor activates signaling pathways that induce proliferation [66].

Three decades ago, the detection of HA on the surface of newly divided keratinocytes firstly indicated a potential link between elevated HA production and cell division [67]. Indeed, another study incubated in vitro rat epidermis with EGF (Epidermal Growth Factor) to induce keratinocyte proliferation and reported simultaneous increased HA production [60]. Consistent with those studies, topical application of HA bound to phosphatidylethanolamine on aged mouse skin (allowing HA diffusion through the hydrophobic cornified barrier) was found able to induce keratinocyte proliferation and associated epidermal thickening [68]. Moreover, injection of HA into a human cutaneous wound model was shown to accelerate re-epithelialization without altering inflammation [69]. When keratinocytes are treated with 4-methylumbelliferone (4-MU), an inhibitor of HA synthesis, an expected decrease in HA production was found in this cell type, accompanied by inhibited proliferation and decreased cell migration related to perturbed cytoskeleton [70]. However, our group more recently observed that 4-MU was likely not suitable to investigate keratinocyte proliferation since the inhibitor affects this process in a HA-independent manner [71]. Despite this limitation, studies have demonstrated some involvement of HA in cell proliferation without the use of 4-MU. Notably, addition of exogenous HA to an organotypic tissue culture provoked an induction of keratinocyte proliferation and related epidermal thickening. In addition, expression of some molecules involved in the epidermal-dermal junction was also reported as increased by the addition of HA [72]. More recently, a treatment with 1-ethyl-β-N-acetylglucosamine (β-NAG2), a substrate converted into UDP-N-acetylglucosamine by cellular metabolism, was found to stimulate HA production in a cultured epidermis, together with increased proliferation of basal cells [73].

However, despite many studies that indicated links between HA production and keratinocyte proliferation, our effort to identify a causal link between these two events remained unsuccessful. Indeed, our group reported that neither a treatment of reconstructed epidermis with an exogenous hyaluronidase activity from a *Streptomyces* bacterium (StrepH), nor an inhibited HA synthesis caused by knocking down *UGDH* gene expression (UDP-glucose 6-dehydrogenase) in keratinocytes produced any significant consequences on keratinocyte proliferation [71]. In summary, the eventual ability of HA to directly modulate keratinocyte proliferation is a question that remains controversial. 

#### 3.4.2. Regulation of Keratinocyte Differentiation

When studying the role of HA in epidermal differentiation, studies have reported HA as being a repressor of the process. In 2003, for instance, a study carried out on rat keratinocytes illustrated that treatment of those cells with KGF (Keratinocyte Growth Factor) was inducing an accumulation of HA by increased HAS2 and HAS3 expression. Simultaneously, the expression levels for differentiation markers, such as the keratin 10 or filaggrin, were conversely reduced [74]. When reconstructed rat epidermis were treated with the exogenous StrepH hyaluronidase, the expression of keratin 10 and filaggrin were found increased as a consequence of HA degradation [63]. Still, another study using StrepH on mouse epidermis to degrade HA led to similar conclusions as the treatment with StrepH was reducing the epidermal thickness by half its normal value. In such conditions, the expression of keratin 10 and filaggrin appear higher than in tissues cultured with intact HA [75]. Those results can be explained if keratinocytes treated with exogenous hyaluronidase are committed earlier towards terminal differentiation, impeding to reach the normal tissue thickness during epidermal reconstruction.

Conversely, other studies have puzzlingly demonstrated an activating effect of HA on epidermal differentiation. However, since different sizes of HA polymers may induce different, or even opposite effects on signaling pathways, HA of different sizes may differentially regulate differentiation of epidermal keratinocytes. An increase in HA amounts production was found to trigger expression of markers, such as claudin and occludin, involved in the formation of tight junctions in differentiated granular keratinocytes [76]. Experiments studying human epidermis treated with 1-ethyl-β-N-acetylglucosamine (β-NAG2) indicated, with increased HA production, some concomitant increase in filaggrin and transglutaminase 1 expression. However, this accelerated differentiation can be slowed down to basal levels by blocking the β-NAG2 substrate converting enzyme [73]. 

Interestingly, the stimulation of differentiation by HA seems also mediated by its interaction with the CD44 receptor. Bourguignon et al. (2004) demonstrated that HA-CD44 interaction was involved in cytoskeletal functions that are essential for keratinocyte adherence and differentiation [77]. Moreover, the addition of exogenous HA was shown to increase the expression of keratin 10, involucrin, and profilaggrin under the dependence of CD44 receptor [78], and the absence of CD44 in the epidermis consistently resulted in decreased keratin 10 and filaggrin expression [79]. Again, another study performed regarding the addition of small HA (tetra-saccharide) molecules to keratinocyte cultures has reported an increased expression of keratin 10 when phosphorylation of CD44 and increased cytosolic calcium concentration happen [80].

Finally, studies conducted by Malaisse et al. (2016) in our laboratory concluded that HA production by keratinocytes and their differentiation process are independent events. Indeed, human reconstructed epidermis incubated with 4-MU or StrepH reveal no impact on the expression of either keratin 10, filaggrin, or involucrin. In addition, even further, keratinocytes deficient for the UGDH enzyme reveal no alteration in their differentiation process [71]. In summary, the relation between HA and keratinocyte differentiation is once again not yet elucidated.

To conclude, possible roles of HA during keratinocyte proliferation or differentiation and their overall importance for epidermal physiology are still a matter of debate. However, although the role of HA in keratinocyte proliferation remains unresolved, its CD44 receptor is a marker of cell stemness [81]. CD44 is a marker for cancer stem cells in several epithelia, but also of keratinocytes enriched for epidermal stem cells with long-term repopulating ability. On the other hand, the maintenance of stem cells and their property for self-renewal are favored when the environment is enriched in HA [82], suggesting involvement of HA and CD44 in the maintenance of an epidermal stem cell character.

#### 3.4.3. Involvement in the Epidermal Barrier Settings and Integrity

The final step in keratinocyte differentiation is keratinization, which means production of the impermeable cornified layer, the most superficial layer of the epidermis. During this late stage of epidermal differentiation, cells become embedded in a lipid ceramide-rich matrix which seals intercellular spaces between the fully differentiated cornified keratinocytes. This process maintains a waterproof barrier on the skin surface [83]. During this process, as early as in the granular layer, lamellar bodies elaborated by the Golgi apparatus are triggered to fuse with the apical plasma membrane of keratinocytes and discharge their content (e.g., sphingomyelin, glycosphingolipids, phospholipids, cholesterol), as well as several acid hydrolases (serine protease, phospholipase A2, sphingomyelinase, β-glucocerebrosidase) in the extracellular matrix, ready to cleave substrates at low pH [84,85]. Between the shedding of lamellar bodies in the *stratum granulosum* and the establishment of a dense matrix in the *stratum corneum*, lipids undergo maturation, thanks to enzymes released jointly by the lamellar bodies. These enzymes are indeed involved in the cleavage of glycosphingolipids into ceramides, and phospholipids into fatty acids. Ceramides account for 50% of *stratum corneum* lipids and are covalently bound to envelope proteins, mainly to involucrin [86]. Cholesterol is another major matrix constituent and accounts for 25% of matrix lipids [86,87]. HA and its CD44 receptor are involved in the establishment of this lipid barrier.

HA and CD44 are involved in formation of lamellar bodies and in the polarization of their secretion. In Cd44 knock-out mice, content and polarized secretion of lamellar bodies are clearly disturbed [78]. While, in normal conditions, lipids are exclusively secreted at the apical pole of granular layer cells, at the interface with the cornified cells, the absence of Cd44 results in dual apical and basolateral secretion of lamellar bodies, indicating unpolarized secretion. In addition, HA-mediated cholesterol synthesis is blocked in those conditions. Indeed, whereas HA increases cholesterol concentration in the epidermis, Cd44^-/-^ keratinocytes treated with HA do not produce cholesterol. As a major consequence, defects in lipid production and polarized secretion impair the formation of the epidermal barrier [78]. In agreement with those observations, the addition of small HA molecules (tetra-saccharide) increases the expression level of enzymes responsible for ceramide synthesis, as well as the intracellular ceramide-content in human keratinocytes in vitro. This HA-mediated enhancement of lipid content improves the water retention in *stratum corneum* of mice following cutaneous UVA irradiation, as assessed by decreased transepithelial water loss [88]. The absence of CD44 also impacts the organization and, thus, the function of epidermal tight junctions. During development, Cd44^-/-^ mice exhibit on day 18.5 of embryogenesis a transient 1-day delay in skin barrier establishment. Such a delay corresponds with alteration in availability and organization of tight junction proteins [89]. In summary, the HA-CD44 interaction is involved in the maintenance of the epidermal barrier integrity and both molecules influence each other. HA influences CD44 expression [90]. Conversely, in the absence of CD44, a smaller amount of HA is detected in the epidermis, which then appears thinner [78].

### 3.5. Epidermal Hyaladherins

In the ECM, HA is able to interact through non-covalent binding with a large number of molecules called hyaladherins or HA-binding proteins. These HA partners are extracellularly localized or attached to the cell membrane [21]. Among cellular partners, the best known are the CD44 receptor and the RHAMM receptor.

CD44 (Cluster of Differentiation 44) is a 19 exons-encoded transmembrane glycoprotein which exhibits numerous isoforms produced as a consequence of alternative splicing and post-translational modifications [6] that modulate CD44 affinity for HA [91,92]. In the epidermis, 18 different transcripts encoding CD44 isoforms have been identified [58]. All variants of CD44 share an extracellular domain able to bind HA, named LINK domain. Other molecules, such as GAGs (heparan sulfate, dermatan sulfate, chondroitin sulfate), are also bound by CD44 receptors, depending on the type of isoforms. GAG-CD44 interaction has been found able to enhance the presentation of growth factors to their specific receptors [58]. HA-CD44 interaction allows the formation of a pericellular matrix [93] involved in the regulation of keratinocyte differentiation [6] and in the development of skin barrier [89]. Actually, HA-CD44 binding induces several various signaling pathways, depending on the size of HA polymers. Indeed, high molecular weight HA molecules bind to the CD44 receptor by mostly irreversible hydrogen bonds and activate RhoA-like RhoGTPase signaling pathways that lead to cell proliferation and migration. In contrast, low molecular weight HA form weaker, more reversible hydrogen bonds, and activate Rac1-like pathways that favor cell adhesion and differentiation [6,34]. The variable effects produced by different HA molecules that exhibit different molecular weight were recently presented in detail and reviewed by Tavianatou et al., 2019 [34].

RHAMM (Receptor for HA-mediated mobility) is another HA receptor characterized by several isoforms that localize at the cell surface, in the cytoplasm, in the nucleus, and even in the ECM. Poorly expressed in physiological conditions, RHAMM receptor is overexpressed in cancer cells and in inflammatory conditions, such as during skin scarring. This receptor is able to regulate keratinocyte migration. Indeed, in Rhamm^-/-^ mice, the migration of keratinocytes during wound healing was considerably disturbed [94].

#### The Case of Epidermal TSG-6 Protein

In the category of extracellular hyaladherins, there exist some proteoglycans, especially versican in the epidermis, but we will emphasize here an anti-inflammatory protein named TSG-6 (TNFα stimulated gene 6). This TSG-6 protein harbors a LINK domain very similar to the LINK domain characterized in the CD44 receptor and responsible for HA-binding [95]. However, interactions between HA and the TSG-6 protein appear stronger than those observed between HA and the CD44 receptor [96]. Interestingly though, binding HA to the TSG-6 protein increases the affinity of HA for its CD44 receptor [97,98]. A possible explanation can be found in the observation that HA binding induces dimerization of TSG-6 protein, causing cross-linking of HA chains, matrix condensation, and increased physical proximity of HA polymers to CD44 receptors [99]. Cross-linking of HA chains and their consequences on the organization of the ECM further occur through inter-α-trypsin inhibitor (ITI), a protein with an even higher affinity for HA than TSG-6. ITI is a plasma glycoprotein belonging to a family of protease inhibitors composed of two heavy chains (HC) linked by ester bonds to chondroitin sulfate and to a bikunin. When ITI localizes at the same site as HA and TSG-6 protein, it can catalyze the formation of a covalent HC-HA bond. This reaction takes place in two steps. Firstly, one ITI heavy chain is transferred to TSG-6 protein by a first transesterification reaction. Secondly, the heavy chain is transferred to an acetylglucosamine residue of HA [100]. Such HC-HA complexes form a more widespread and hydrated HA network than the network resulting from HA-TSG-6 complexes [97]. Six heavy chains have been identified, and HC1 and HC2 have been detected in the normal epidermis [101]. The predominantly expressed heavy chain in human skin (at the dermal level) is HC5, whose expression is increased in inflammatory skin conditions, such as allergic contact dermatitis [102]. HC5 has been shown to bind and stabilize HA, and to participate in matrix formation [103]. The TSG-6 protein, although possessing anti-inflammatory properties, is expressed and secreted in the epidermis under physiological conditions [101]. We recently found that the epidermal TSG-6 protein is involved in the retention of HA between keratinocytes, in the thin intercellular spaces between epidermal cells [65]. The HA maintained between keratinocytes by TSG-6 protein creates space and reduces cohesion between keratinocytes, allowing the diffusion of cytokines and growth factors [104]. Furthermore, the TSG-6 protein is widely expressed and secreted in skin under inflammatory conditions related to a proper epidermal wound healing [105,106]. It is also secreted by keratinocytes in response to epidermal infection by dermatophytes [107], or when epidermal cells are challenged by Th2-immune conditions [65,108]. Simultaneously, HA is massively produced in inflammatory conditions resulting in increased intercellular spaces in which cells can migrate [104]. Of course, the TSG-6 protein is expressed and secreted by many other cell types than epidermal keratinocytes. It may, therefore, play other roles than those discussed in the epidermis, as it is detailed by Day and Milner, in their recent 2019 review on this protein and its function [22].

## 4. Epidermal HA in Atopic Dermatitis

### 4.1. Atopic Dermatitis

As one well-characterized pathological condition observed in skin, atopic dermatitis can exemplify how much HA can be involved when some abnormal tissue conditions occur. Atopic dermatitis (AD) is a chronic inflammatory skin disease characterized by xerosis, spongiosis, and hypogranulosis, as well as altered skin barrier [109]. The etiology is controversial since many factors are involved: environmental factors, genetic predisposition, defective barrier, and immune dysregulation [110]. There are two main hypotheses proposed regarding the development of AD. According to the “outside-inside” hypothesis, patients have a defective epidermal barrier that potentially allows allergens and/or pathogens entry in the skin, leading to inflammatory and immune responses. Conversely, the “inside-outside” hypothesis suggests that a preexisting immune dysregulation initially impacts the barrier. Indeed, any triggered release of Th2 cytokines (such as interleukins 4, 13, 25) exhibits proven ability to weaken the epidermal barrier. In respect to both hypotheses, the situation created during atopic dermatitis actually leads to some vicious circle which reinforces the diseased state [19]. Available review papers, e.g., De Vuyst et al., 2017 [111], and Huet et al., 2018 [112], propose models to study atopic dermatitis based on in vitro reconstructed human epidermis. The inside-outside condition can be mimicked by creating inflammatory conditions in the models, for instance, when RHE are incubated with mixtures of cytokines typically elevated in AD (IL-4, -13, -31, -22, -25, and/or TNFα). The presence of Th2 cytokines results in decreased expression of late differentiation markers, such as keratin 10, filaggrin, involucrin, and loricrin, along with a reduction in ceramides and free fatty acids content. Together, such alterations in the epidermal differentiation program that is responsible to create and maintain the epidermal barrier contribute to the impairment of its function. According to the outside-inside hypothesis, genetic factors in this case may be involved in barrier defects. In favor of this hypothesis, RHE produced by filaggrin-deficient keratinocytes display increased permeability and increased sensitivity to Th2 cytokines treatment. In addition, many other environmental factors, such as abuse of detergent products on the skin, affect the lipid-based epidermal barrier in vivo [110].

### 4.2. Implication of HA in Atopic Dermatitis

**Figure 3:**
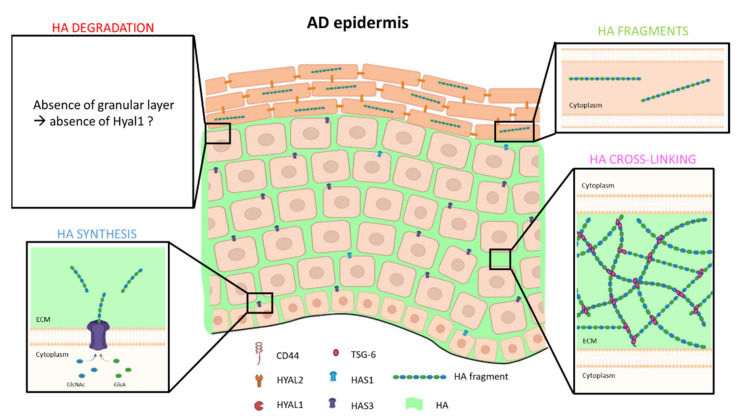
Model of HA metabolism in atopic dermatitis epidermis. Low molecular weight HA fragments are synthetized and extruded by HAS3 enzyme in ECM of basal and spinous layers, while HAS1 mRNA is less expressed. In inflammatory conditions, TSG-6 protein is more expressed in parallel with an increase HA production, allowing a strong cross-linking. As atopic dermatitis is characterized by hypogranulosis, expression and activity of HYAL1 are probably disturbed, and HA fragments are found in corneocytes.

Remaining low in normal conditions, the mRNA expression of *HAS3* has been reported as upregulated in AD skin conditions, simultaneously with well-recognized AD markers, i.e., *CA2* and *NELL2* [29,113]. Indeed, differences in *HAS* expression profiles have been reported between healthy epidermis, mainly expressing *HAS1* mRNA, and the epidermis of AD patients where elevated *HAS3* expression is found (*HAS2* remains scarcely expressed in adult epidermis) [29] (Figure 3). Similarly, when inflammatory conditions related to UVB exposure are imposed to keratinocytes, *HAS3* mRNA expression is upregulated [36,114], and *HAS3* mRNA is also overexpressed upon stimulation by interferon γ, whilst diminished following TGFβ challenge [115]. Consistently, HAS3 enzyme exhibits high activity and produces low molecular weight HA fragments [27]. Such low molecular weight fragments participate to tissue inflammation because they behave in the epidermis as DAMPs (Damage Associated Molecular Pattern) and bind Toll-Like Receptor (TLR) 2 and TLR 4 (for more details, see the review by Tavianatou et al., 2019 [34]). TLRs, through activation of NFκB, are the initiators of release of pro-inflammatory cytokines, as well as inducers of *HAS3* overexpression, thereby amplifying local synthesis of low molecular weight HA and reinforcing the inflammatory response as a consequence [116]. In addition, the heparin-binding EGF-like growth factor (HB-EGF) released from keratinocytes in atopic dermatitis also increases *HAS3* expression [29,117].

Overexpression of *HAS3* in a model mimicking atopic dermatitis causes massive accumulation of HA in the intercellular spaces between keratinocytes of the living epidermal layers [19]. Increase in the amount of intercellular HA is consistent with enhanced detection of HA in ECM of severe eczema skin in parallel with HAS3 overexpression [118]. Accumulation of HA in intercellular spaces can also be related to spongiosis, typical of AD skin. In severe eczema, some reduced expression of epidermal cadherins results in water entry in the tissue and spongiosis [118]. The presence of HA in larger amounts than in normal skin might be responsible for water retention between keratinocytes and for spongiosis. Alternatively, the enlargement of intercellular spaces due to reduced cadherin-driven junctions might favor accumulation of HA in the available space. Of interest, as HA is particularly retained in intercellular spaces by TSG-6 protein [65] (Figure 3), and TSG-6 protein is increasingly produced in vivo in AD skin, such as in vitro in reconstructed epidermis challenged with Th2 cytokines, this anti-inflammatory protein might favor the accumulation of HA in pathologies, such as AD [65]. 

In inflammatory epidermis, overproduction of HA is accompanied by overexpression of the CD44 receptor. Abundance of this receptor is thought to play a role in recapture of HA released in excess, in order to finally reduce and resolve inflammation [35]. In parallel with HA endocytosis, hyaluronidases 1 and 2 enzymes degrade HA, according to the model proposed by Tammi et al., 2001 [57]. 

AD is an inflammatory condition characterized by an absent granular layer, where HYAL1 enzyme is located and active in physiological conditions [51]. According to this model, HYAL1 may be less active in AD lesional epidermis (Figure 3). In line with this hypothesis, Hyal1^-/-^ mouse epidermis is characterized by high-molecular-weight HA contained in cornified keratinocytes [51], whereas solely fragments below 6 × 10^4^ Daltons are detected in cornified cells of wild-type epidermis [20]. Consistently, the typical parakeratotic superficial layer during contact dermatitis contains HA of molecular weight superior than 6 × 10^4^ Daltons [119]. While low molecular weight fragments would contribute to hydration, such as NMF [20], high molecular weight HA in excess in the cornified layer would have a negative impact on properties of this layer and, therefore, on its barrier function. Indeed, high molecular weight HA are able to efficiently retain larger overall content of water in comparison with small fragments of HA, counteracting the formation of the normally hydrophobic cornified layer and leading to some potentially increased epidermal permeability.

In epidermal ECM, HA can interact with other matrix components. Notably, versican, a proteoglycan secreted in the ECM of the basal layer of epidermis, is able to interact with HA [12,120]. This proteoglycan is involved in the formation of a provisional matrix required by leukocytes during inflammatory response, such as the one observed in AD [23]. Decorin, another proteoglycan, is secreted between cells of the suprabasal layers of the epidermis [121]. Decorin mRNA expression was significantly decreased when the HA extracellular matrix is disorganized, due to the lack of the TSG-6 protein [65]. In contact dermatitis, decorin-deficient mice exhibit a weaker inflammatory response than wildtype mice as a consequence of impaired leukocyte recruitment [122]. Those studies highlight the importance of the organization of the epidermal matrix composed of GAGs (HA) and proteoglycans (versican and decorin) in the regulated recruitment of immune cells in an inflammatory context. Moreover, when HA chains are properly cross-linked and organized by the TSG-6 protein, certain pro-inflammatory growth factors and cytokines (such as IL-8) can be sequestered [123,124], thereby limiting the presentation of the ligands to their respective receptors and, thus, regulating the attraction of neutrophils toward the inflamed site [106]. 

Atopic dermatitis is characterized by hyperplasia. Epidermal thickening is apparently a consequence of overexpressed EGF receptor (EGFR) [125] and HB-EGF [29,126]. HB-EGF is rapidly cleaved and available as a soluble ligand in the ECM during the inflammatory setting [127]. HB-EGF can then bind EGFR and activate downstream pathways that can induce epidermal proliferation and elevated *HAS3* mRNA expression [29,117,128]. In parallel, stimulation of EGFR induces the expression of pro-inflammatory cytokines, such as IL-8, that recruit leukocytes [125]. Interestingly, EGFR is localized in plasma membranes cholesterol-rich lipid rafts, close to CD44 [60]. It has been shown, in cancer cell lines, that EGFR and CD44 receptors interact to auto-regulate HA synthesis [117]. 

In summary, HA and other ECM components (proteoglycans, cytokines, growth factors) appear to support HA production in order to generate an organized matrix for the efficient and controlled recruitment of inflammatory cells in cutaneous pathologies.

## 5. Conclusions

HA is a glycosaminoglycan found in intercellular spaces. Although secreted in many vertebrate tissues, 50% of HA is localized in the skin. Predominantly detected in the dermis, HA is, nonetheless, also located between keratinocytes in living layers of the epidermis, where it appears involved in development and maintenance of a competent epidermal barrier. In addition, HA plays controversial roles in proliferation and differentiation of epidermal keratinocytes. The enzyme HAS1 seems mainly responsible for the production of HA in the epidermis in normal conditions, but a switch in expression of the different HAS enzymes in the pathological epidermis, characterized by overexpressed HAS3, alters the molecular characteristics of epidermal HA in skin diseases. For its degradation, HA is processed via a CD44-HYAL2-HYAL1 axis, suggesting that HA is potentially internalized and degraded into very low molecular weight fragments when it reaches the granular cell layer of the epidermis. This review discusses the recently noticed involvement of HA and its metabolism in barrier alteration-related diseases, such as atopic dermatitis. Further studies are still awaited to fully understand the role played by HA in the epidermis, either under physiological conditions, but also in the context of skin diseases.
